# Twenty-five Years of Research Experience with the Sterile Insect Technique and Area-Wide Management of Codling Moth, *Cydia pomonella* (L.), in Canada

**DOI:** 10.3390/insects10090292

**Published:** 2019-09-10

**Authors:** Howard M. A. Thistlewood, Gary J. R. Judd

**Affiliations:** 1 Summerland Research and Development Centre, Agriculture and Agri-Food Canada, Summerland, BC V0H 1Z0, Canada; 2 Department of Biology, University of British Columbia, 3333 University Way, Kelowna, BC V1V 1V7, Canada

**Keywords:** Lepidoptera, *Cydia pomonella*, mass rearing, quality control, overflooding, area-wide management, sterile insect technique, mating disruption, pheromone, fruit damage, ecology, diapause

## Abstract

The advent of novel genetic methods has led to renewed interest in the sterile insect technique (SIT) for management of insect pests, owing to applications in mass rearing and in the production of sterile offspring without use of irradiation. An area-wide management programme for codling moth, *Cydia pomonella*, has employed the SIT and other management practices over a large area (3395 to 7331 ha) of orchards and neighbouring urban, public, or First Nations lands in British Columbia, Canada, for 25 years. This project is the first to employ the SIT for *C. pomonella*, and the longest-running application of area-wide techniques for its control, anywhere. It was derived from basic research and applied trials from the 1960s onwards. Many biological challenges were overcome, and lessons learnt, in transferring from small- to large-scale applications of mass rearing and the SIT, with particular regard to Lepidoptera. Research has proven essential to identifying, if not resolving, issues that threaten the implementation and success of any such programme. The major challenges encountered, and the resulting research, are reviewed, as well as future directions. Recommendations are given for application of the SIT as part of any area-wide management programme for *C. pomonella*.

## 1. Introduction

The Okanagan-Kootenay Sterile Insect Release (OK SIR) program is the longest-running application of area-wide management for codling moth, *Cydia pomonella* (L.), in the world, and the first to employ the sterile insect technique (SIT) for its control. It is a unique creation of co-operating regional governments in the main tree fruit-growing area of British Columbia (B.C.), Canada. It has operated since 1992 under provincially legislated authority and in consultation with First Nations on their lands [1]. Services that reduce the population of *C. pomonella*, including host tree removal, insecticide use, release of sterile moths, pheromone-based mating disruption, tree banding, fruit stripping, legislation, mapping, public relations, and education, have been provided. Currently, it acts over a service area of 21,000 km^2^ in some 3520 ha (formerly 7331 ha in 1992) of conventional and certified organic pome fruit orchards, and in >24,000 (circa 40,000 in 1992) noncommercial sites located in parks, gardens, abandoned properties, and road-sides [2,3,4].

Key elements of the OK SIR program arose from the work of entomologists since the early 1900s on the relatively small pest complex in the mountainous interior of B.C., which resulted, in part, from its geographic isolation and an early imposition of quarantine restrictions (reviewed by Dendy [5]), as well as a semi-arid climate. The codling moth was discovered in the region in 1905 and was the subject of repeated attempts at eradication, until such efforts were abandoned in 1925 [5], after which it became fully established in all pome fruit sites and the key pest of apples [6]. It has two full generations (spring, summer) and a partial third in the region, and overwinters as diapausing fifth instar larvae on the wood of host trees, around the base of trees, or on wooden objects nearby. Concerned with the impact of insecticides being applied for a small number of key pests, M.D. (Jinx) Proverbs focused on the possible use of the SIT as a highly selective management method for *C. pomonella* in the Similkameen Valley of B.C. (Section 2). Proverbs showed that codling moth populations in a group of contiguous orchards could be reduced to undetectable levels for one or more years after its application, under certain conditions, during 1965-1980. Ten years later, the designers of the program had extended his ideas into an eradication project operating in two very different regions and several valleys, ignored viable alternatives [7], and had “tinkered” [5] with many core elements to suit economic and political agendas. Many scientists at the time repeatedly cited little or no need for further research for success (e.g., [5,8,9]). Scientific and economic reviews (see [9,10]) supported the concept, in general, with a few caveats and some recommendations for changes to plans, timelines, or cost estimates (Section 2 and Section 3.1).

In reality, a series of problems led to a recurring inability to predict timelines toward results, and regularly forced changes in operations, cost estimates, and overall goals [2,3,11]. The major ones are described in the present article, with solutions or directions taken. It is now accepted that an area-wide programme cannot apply SIT successfully without continuing research and without extensive reliance on other techniques [12]. However, despite public investment exceeding some C$85–100 million to date, few publications describe the entomological challenges of the B.C. project, which are important lessons [13]. Attention has been given to improved understanding of the rearing and radiation biology of sterile moths, but less to other biological questions, or other technologies, and their integration in a large programme. The year 2019 marks 25 years since the start of rearing and release operations and 50 years since a major review by Proverbs [14]. In this review, we summarize the main research challenges experienced since the OK SIR program began in 1992, and the research in response to such factors, primarily from the Pacific Northwest of North America. We believe this example to be valuable more generally, because new genetic techniques are being suggested for application of the SIT to many insects, often without regard to important behavioural and ecological factors. We follow Proverbs [15], who emphasised the need for improved knowledge of population processes at the time when SIT was first under consideration for many insects.

## 2. Historical Background

The major functions of an operational SIT programme can be divided into three areas of research: (1) Mass rearing of insects and quality control of production, (2) effects of sterilization and handling on viability and field performance of released insects, and (3) application of insects to the target population, and the associated monitoring of results. Over a 20-year period before the OK SIR program was planned, these elements were developed from theory, by applied research, or adapted from experience with very different insects [15,16,17]. Such a programme required many novel elements, including a system of mass rearing using artificial diet [18]; a rotating oviposition chamber [19]; automation and mechanized equipment, including for moth release [20]; a means of distinguishing released insects in the field [21]; and assessment of quality control using genetic markers [22]. Concurrently, experiments in the then-novel technology of irradiation clarified aspects of the biology of sterile codling moths [23,24,25,26], and of the relative viability and competitiveness of wild and irradiated insects [27]. Once relatively large numbers of sterile moths could be reared, the possibilities of field application for seasonal control or local eradication were explored in many experiments [14,17,28,29,30,31]. An increasingly large and complex set of field experiments [31,32,33] led finally to a pilot study of SIT in 320–526 ha of adjacent orchards in the Similkameen Valley from 1975–1978 [34].

The success achieved with research into SIT for *C. pomonella* led to consideration of more general use internationally, particularly because of growing interest in area-wide programmes (e.g., [35]). By 1980, interest in the SIT approach had ceased elsewhere, for reasons including increased attention to the abundance of untreated host trees in many regions, cost, and because of the development of pheromone-based mating disruption [15,16,36,37,38,39,40]. Meanwhile, in B.C., a proposal was developed for a codling moth pest management programme employing SIT and using locally proven methods. It turned into an eradication programme using SIT, largely for reasons of costs, economies of scale, and politics [5,10,16,17,41,42]. Partial accounts of the biological challenges, research, and results were reported to 2005; mostly in conference papers or non-reviewed sources [5,7,11,13,43,44,45,46,47,48,49,50,51,52], and a simplified history follows (additional document locations are provided in Table S1).

The OK SIR program began in 1992 under a plan to divide the service area, comprising a series of interior valleys, into two zones, North and Creston Valley, and South. By splitting the eradication into two successive phases or zones, the resources required, particularly sterile moth supply and associated size of mass rearing facility, were reduced greatly. It has operated in three modes to date: rapid eradication (1992–1996), slow eradication (1996–1999), and area-wide management (since 2000). Under the rapid eradication concept, intensive use of insecticides was necessary to reduce codling moth populations to levels suitable for SIT to work, which were defined as apple harvest damage levels of 0.1%–0.3% prior to release. It was expected that *C. pomonella* would be eradicated within three years per zone, or six years in total, at a cost of C$12 million [8,9]. The number of sterile moths required for the service area was estimated using a minimal ratio of sterile–wild moths at each location [5,8]. A simple mathematical model suggested that an average sterile–wild ratio of 40:1 in the first of two generations would lead to extinction of a wild population across some 9000 ha within three successive generations, i.e., 1.5 years, per zone [9].

By 1996, despite intense efforts, multiple problems had resulted in the same proportion of sites having codling moth populations as in 1994 [3,53]. Operational experience had doubled the estimated time for eradication, caused the program to split the service area into three zones, and quadrupled the projected costs [7,54]. Contrary to predictions [44,45] and a brief technical review at the time [7], a detailed analysis [53] in 1998 revealed that eradication was not occurring. By 2000, the program was restructured as an area-wide management project using multiple technologies [11]. Program goals were changed to (1) reduce insecticide use, (2) to reduce damage from *C. pomonella* to a low level (0.2% fruit damage in 90% of orchards) throughout the entire service area by 2005, and (3) to achieve a low-cost steady state (defined as equivalent to the level of moth presence in the Similkameen Valley, circa 2001 [1,2]) across the service area by 2006 (later delayed to 2008 [1]). The first goal was achieved by 2000, when pheromone-based mating disruption replaced insecticides in difficult regions and years, the second goal was first reached in 2009 and in some years subsequently, and the third goal has not been attained. By 2015, a novel strategic plan [55] no longer mentioned the three goals.

Detailed statistics on performance of the OK SIR program were provided intermittently in technical reviews from 1998–2014 [2]. Subsequently, only weekly maps of wild moth numbers [56] or maps of orchards requiring supplementary use of insecticides or mating disruption [57] have been available. Current release rates are reported as 2000 sterile mixed-sex (1:1 ratio) moths per ha, once per week [4], with total production near 10 million per week, 96% of which is released or sold to external users in the USA and internationally [58]. Some pheromone-based mating disruption and/or cardboard tree banding is deployed in limited situations. It is recommended that orchardists apply supplemental treatments (e.g., insecticides, granulovirus, mating disruption) during spring in orchards with wild moths [57], or when moth captures seasonally exceed >2 wild moths per trap for two weeks consecutively [59]. Alarmingly, the number of orchards in both categories has increased in recent years. Both wild moth counts and proportion of sites with codling moth are relatively high at the time of this review [56,57].

Finally, it is usually the economics of a codling moth, or any large project, which determines its destiny [35,60,61,62]. In B.C., the funding is received largely (circa 60%) from local property taxes and from charges per ha of pome fruits to growers [1,4]. The cost is currently C$3.7 million annually to protect 3395 ha [4], for a single pest. The current area-wide programme is the most expensive way to manage codling moth, globally, at near $1120 per ha. The SIT component has always been the most expensive in prior economic reviews [2,8,10,41,42,63,64]. Additional costs are borne by growers for supplemental controls required at certain population levels (insecticide, mating disruption, etc.) [57]. The overall economics have been justified by claims of environmental savings in reduced insecticide use, and other community benefits [1,4,8,65]. Those aspects are beyond the scope of this article but, unfortunately for the current review, no evidence for such benefits, nor any detailed data or program assessment, has been available since at least 2014 [2].

## 3. Research Areas Arising from Operational Challenges

### 3.1. Mass Rearing Strategy, Handling, and Quality Control

The quality of sterile male insects when released is critical to any effective project using the SIT [66]. The mass rearing process for *C. pomonella*, as employed in B.C., is approximately as summarized by Dyck [67]. Adult moths oviposit onto wax paper in cylindrical rotating cages and the egg-laden paper is cut and laid onto trays of a saw-dust based diet in temperature-controlled rooms. The eggs hatch and larvae develop whilst ingesting a permanent internal dye marker. The trays are later placed in emergence rooms, the adults emerge and fly towards an ultraviolet lamp in the ceilings, where air movement sucks them gently into a collecting system, which deposits them in bins in a cold room. The adults are packed in plastic Petri dishes, irradiated, and returned to cold storage until distributed for release. Moth release occurs within marked rows of pome fruits in orchards, using all-terrain quad vehicles. On the front of each vehicle is mounted a mechanical hopper, which drops moths onto a horizontal moving belt, from where they are blown gently by a fan onto the orchard floor.

High levels of contamination with a *Cydia pomonella* granulovirus (CpGV) were a problem in the early years, owing in part to building design or layout [3,68], and contributed to poor field performance at the time [69]. A rapid test for virus occurrence was developed to monitor its frequency within the colony and in the wild population (23% of samples) [70]. Continuing studies of virus presence in the rearing facility led to improved quality and minimized the effects of pathogens in mass-reared insects [68]. A rigorous application of hygiene and standard operating procedures has, in recent years, kept CpGV levels to a minimum, except when the colony is stressed [71]. Current research seeks to identify the strain(s) of CpGV occurring in this mass-reared colony and in local field populations [71]. It also has potential applications in monitoring and management of any more virulent strains [72] that may be introduced to the region.

Assessments of insect quality should include measures of the sterile moths both in mass rearing, and in the field after release. Before the program started, external reviewers were concerned that insufficient tests were planned for quality assurance [10]. This continues; for example, major behavioural changes in mass-reared program moths were measured in 1996, 1998, and 2002–2004 [48,71], as were other such changes reported from codling moth facilities elsewhere [39,73,74]. In addition, Canadian and American users of mass-reared sterile moths from the OK SIR program have reported a distinctive behaviour in sterile males over a 20-year period [71]. A large proportion of the latter are regularly captured within pheromone traps in the reverse orientation to wild males, i.e., glued dorsally and not ventrally. Despite such differences, the standard assays used for other insects in SIT projects have not yet been developed. Improvements in field performance of traps and lures are reported in Section 3.8.

In order to avoid such problems, Proverbs kept laboratory colonies in mass rearing for no more than two years at a time [38], which was noted for contributing to the success of his work [34,39]. To date, only limited efforts to replenish the program colony have been reported [2,3]. At the time of this review it has not been augmented with wild moths for four years [71]. A recent collaborative research project (CRP) on SIT for *C. pomonella* [75,76] provided laboratory measures of mobility [77,78,79], including a flight cylinder test [80], which may be more easily adopted for quality assurance in future. However, the results found in field cages differed from those in the laboratory [81], as was similarly reported in a different study that compared flight tunnel and field assays [82]. The CRP also demonstrated the viability of shipping program moths internationally [83,84], and earlier work on mating compatibility of populations from different parts of the world was confirmed [85,86].

Given the climatic requirements of pome fruit production, a major concern was always to optimize the rearing capacity and staff of a facility able to rear moths year-round but releasing during only five months of the year. From the beginning, ways of stockpiling moths were investigated by rearing into diapause conditions and holding for subsequent emergence and use in the growing season [87]. In so doing, important competitive differences were identified between mass-reared moths and laboratory-induced or field-collected diapause moths, or between moths subject to different handling regimes [69,82,87,88,89,90,91]. Unfortunately, the most recent summary [91] concluded, on the basis of field trials, that introduction of a diapause phase into the mass rearing system cannot be recommended for increasing sterile–wild mating, nor for improving trap-measured ratios of sterile to wild males (Section 3.8).

The effects of rearing *C. pomonella* under different temperature conditions were also studied in laboratory or field comparisons [78,82,91,92]. Rearing under fluctuating temperatures regularly provided moths that were significantly more competitive than those reared under constant temperature or through diapause. To date, the purpose-built facility has been unable to achieve such different thermal requirements at the same time as responding to the external changes in seasonal temperatures experienced in Osoyoos, B.C. [2]. Research into the effects of handling practices has proven that significant improvements in the quality of sterile moths and increases in sterile–wild ratios can be gained by changes in the collection and delivery system [77,82,91]. It is recommended that the time in cold storage should be limited and, consequently, thermal acclimation has been of interest, but results have not yet been easy to interpret or implement. The effect of pre-release chilling on flight capacity was examined and the cold storage procedure as employed in the facility did not always have a negative influence on adult flight capacity in the laboratory [93], but differences (3 h versus 24 h) in chilling time were linked with significant differences in mating competitiveness [91]. In laboratory and field studies in South Africa, it was found that gains in performance seen when using low temperature acclimated moths under cool conditions in the wild came at a cost to performance under warmer conditions [94]. However, a combination of field and laboratory research has shown that significant improvements in competitiveness occur if steps are taken to minimize moth damage (such as loss of wing scales) while being carried, and to dispense moths into the canopy rather than onto the ground, or other changes in the delivery system [82]. Proverbs [16] originally used helicopters for aerial release, after viewing problems in fixed wing application tests in the USA, but settled on ground-based application as more suitable to the terrain in B.C. Presently, aerial release by the use of drone technology is being tested in Canada and the USA.

Most rearing or acclimation studies have employed irradiated mass-reared sterile moths as the standard for measurement of improvement of field performance. Most have not employed wild moths against which to measure any differences, despite the observed differences in results when sterile moths are compared with wild moths [69,91], and the demonstrated importance of such differences [95]. However, two difficulties occur when using fertile wild moths: The gathering of a sufficient quantity from the wild; and the risk of fruit damage when releasing in high-value commercial orchards. Also, many of the field studies cited earlier have used simple measures of relative performance in the field, such as distance flown or male recapture rate, rather than important measures of reproductive success [15]. Note that the measures are usually based on male response to traps baited with synthetic lures (1 mg of codling moth sex pheromone (E,E)-8,10-dodecadien-1-ol, i.e., codlemone) rather than with wild calling females. Such simple measures have been criticized because of their lack of attention to true mate competition [89]. In summary, “…little substantial QC (quality control) work has been done on the codling moth… field measurements are expensive, time consuming and not readily adaptable to routine quality testing” [67]. Studies to improve overall field performance by manipulation of factors other than in rearing are described below.

### 3.2. Radiation Dose, Competitiveness, and Inherited Sterility

The choice of appropriate radiation dose for Lepidoptera is complicated because they are, to a degree, self-repairing from radiation damage. Relatively little guidance can be taken from SIT as used for other insect groups, which generally require much lower and less damaging levels of radiation for sterility than moths [14,24,96]. Moths have a relatively complex genetic system, which is not yet understood in detail for purposes of SIT, and that of *C. pomonella* is still under investigation (e.g., [76]). A distinction of lepidopteran radiation biology is the production of offspring in the F1 generation under some combinations of partially sterile–wild mating. A proportion of F1 larvae may survive to adulthood, and can be partially to fully sterile [17,97,98]. If a wild moth population has reached low enough levels for the SIT to function, it is theoretically possible that the release of large numbers of sterile males treated with a low radiation dose permits survival of a proportion of fruit-damaging larvae and the subsequent production of unmarked sterile F1 moths [17,33,99].

When the program began, the radiation biology of *C. pomonella* was relatively better understood than were other relevant aspects of its biology and ecology [15]. The radiation dose employed had to effect a compromise between the levels of sterility induced in males and females, any damage-producing potential without sterility, and their competitiveness in mating with wild moths [100]. Over time, the minimum radiation dose at which females of *C. pomonella* are found to be sterile has varied, using various systems of irradiation, from estimates of 350–400 Gy [33,96,101], to as little as 100 Gy [102,103] and the resulting level of partial male sterilization has also varied. Initially, for the OK SIR program, irradiation at 250 Gy was recommended [42], resulting in 85%–92% sterility of males, rather than the suggested 98% at 400 Gy, but some scientists insisted on 300 Gy or more [41], in order to avoid damage in commercial orchards. A compromise treatment of 350 Gy was recommended [34], with some fruit damage expected as well as production of F1 moths [9]. The dose employed over time has varied (by year shown): 330 Gy (1994), 320 Gy (1995–1996), 250 Gy (1997–2001), 150 Gy (2002), 250 Gy (2003–2013), and currently is 200 Gy (2014–present) (S. Arthur and L. Tomlin, OK SIR program, pers. comms.). The latter dose is also reported for OK SIR program moths sent to a New Zealand eradication programme [61].

The reductions in dose from 330 to 250 Gy in 1997 were made following studies of radiation dose, inherited sterility, and competitiveness in summer releases or in the laboratory [102,104]. The change to 150 Gy in 2002 was controversial. External consultants pushed for lower radiation doses (100–150 Gy) in the hope of producing inherited sterility in F1 moths, and subsequent fast reduction of the wild population [2]. Owing to resulting problems [2], described below, a return to 250 Gy occurred the next year. Field cage and orchard experiments have tested treatments as low as 100–150 Gy [48,105]. In laboratory studies, treatment with 150 Gy was recommended for use in South Africa [103]. In summary, two reviews of many field experiments with *C. pomonella* concluded that a reduced radiation dose has provided measurable increases in competitiveness of sterile moths under some conditions, but not always, and almost never in the critical spring generation flight period [48,95]. Significant differences can be measured in summer, but result in inconsistent or no differences during the spring generation, the importance of which is discussed below (Section 3.6).

Balancing the effects on competitiveness are practical considerations that are critical in high value crop production when not in eradication mode. For codling moth, there is a relatively fine line between healthy, competitive, but fruit-damaging adults, versus radiation-sick, sterile adults causing no damage [17]. LaChance explained this as follows “for Lepidopteran insects, the dose can be considerably lower than the full sterilizing dose without the risk of adding insects to the natural population. However, two problems arise. Growers may object to the presence of F1 males and females on their crops even if such insects are sterile. Also, the dose administered to the released insects must be carefully adjusted so the progeny of outcrossed (colony) females are not fertile” [98]. Proverbs [14] warned that “with high-priced crops it might be uneconomical to “rear” sterile F1 progeny in the field” and later [106] clarified that “in a control program by sterile insect release, the larval population capable of fruit injury is very important, whereas in an eradication program it is only the larvae that develop into adult moths that are of primary concern”.

In field cages, fruit damage and superficial “sting” damage (skin penetration followed by late embryonic lethality in F1 larvae) occur at many levels of radiation producing F1 moths [17,102]. However, it is clear that we do not understand the circumstances governing moth survival and the level of damage produced at various radiation doses [97,98,106]. These warnings were first understood in 1998 when, in an unusually warm and lengthy season, high levels of superficial “sting” damage occurred on fruit in many orchards receiving 250 Gy moths [71]. It caused a widespread loss of trust in the SIT [2]. Secondly, in regions with comparatively few wild moths, some local damage occurring from F1 progeny can be followed by the presence of unmarked F1 males, complicating interpretation of trap catch and allocation of resources. These effects were reported in one pilot project of codling moth SIT [48,107] and from one of the few other operational SIT projects against moths, i.e., pink bollworm, *Pectinophora gossypiella* (Saunders) [99,108]. Such effects were also widespread in the OK SIR program in 2002, when a dose of 150 Gy was used season-long. Consequently, 250 Gy was reinstated as the dose for the next ten years, until it was reduced to 200 Gy in 2014. It is therefore possible that some of the relatively high numbers of unmarked (normally wild) male moths captured in recent years [56] may be F1 sterile males, given earlier results using 150 Gy. Molecular studies are underway towards development of a technique to distinguish the origin of unmarked males in traps [71].

### 3.3. Overflooding, Synchronicity of Supply, and Mating of Sterile Moths

The achievement of target sterile–wild ratios at each site is critical to the SIT, which works by overwhelming wild moth populations. Proverbs [15] required three important measures of population processes to be determined when operating any SIT programme for *C. pomonella*: The ratio of sterile to fertile male insects (overflooding ratio, sterile–wild), population resilience (change in population density over successive generations), and immigration rates. The first and last of the three measures have received some attention, as discussed here and later (Section 3.4). The importance of achieving the necessary ratio in every part of a treated area was explained in an exhaustive series of field trials [16,17,30,31,32,33,34,38,109,110,111,112]. It was demonstrated that an overflooding ratio of between 8 or 10:1 at a location was insufficient to prevent damage, and that 20:1 may prevent a wild population from increasing but would not eliminate damage. If the control area is small and subject to immigration, as are most B.C. orchards, the minimum ratio at all locations must be 40:1 and only that could induce a downward trend in the wild population. At the same time, researchers in a 3-year study of SIT in Washington State concluded that the reason for local damage at specific trap sites was a failure to continuously exceed a 20:1 sterile–wild ratio at those sites [113]. For population reduction, minimal overflooding ratios of 40:1 were also reported as necessary by others [114], or should be exceeded [89], by as much as 60:1 to 120:1 [41] or 100:1 [61], for attempted eradication.

Supplying sufficient numbers of moths throughout the season to achieve target ratios has proven to be a significant challenge. Despite a change of mandate from eradication, dividing service from two zones into three zones, lengthening timelines, and a significant reduction in service area [2,71]. Initially, target overflooding ratios were not met, owing to inadequate calculations for the necessary moth production and underestimates of size of the wild population [43]. The original concept was for a total of 25,000 moths per ha to be released in twice weekly instalments during each 20 to 25 week season [9]. The latter was a rate emphasised by Proverbs [16] only for use in orchards with <0.05% damage at harvest, whereas the population levels targeted by the program were two to six times higher [9] (Section 2). The number per ha is also much less than the release of up to 36,500 moths per ha used in the pilot project of 500 ha [34], from which the OK SIR program was derived. Also, in all Proverbs’ experimental studies, high sterile–wild ratios were achieved by careful use of moths reassigned daily to locations according to trap captures. This was necessary because of limited supply from his mass rearing colony, as well as a recognised need for mating synchronicity, as discussed below.

The inability to achieve overflooding ratios at many sites was considered to be a major part of the reason for the slow progress of the eradication program, 1994–1998 [2,43]. Detailed analysis of the ratios achieved at every trap from 1994–2003 [48] and in nine or ten commercial orchards from 1995–1999 [115] showed a consistent inability to achieve useful overflooding ratios throughout the spring generation of *C. pomonella* activity and in a high proportion of sites during parts of the summer generation [52]. Consequently, a series of rearing and acclimation studies (Section 3.1) attempted to improve competitiveness and overflooding ratios.

A second important factor is the synchronicity of the sterile and wild moth flights and mating periods, because mating competitiveness is the single most important element of SIT [66]. Results of experiments measuring mating on a daily basis have shown that the wild moths are better synchronised than are colony moths for timing of arrival and mating during wild female calling periods [95]. This is particularly evident during cool spring flight periods, and so an overflooding ratio read from a trap once (or at most twice) weekly may be biologically irrelevant [95]. This results from the scramble competition mating system employed by codling moth, whereby the first male(s) to respond at the time of female calling are the critical ones for mating success. Sterile moths arriving late to a trap may increase the apparent sterile–wild mating ratio, but have little effect upon wild moth control. Furthermore, sterile, mass-reared males from a nondiapause culture released by the OK SIR program lacked mating competitiveness with wild males in spring; <1% of males were able to successfully locate and mate with tethered wild females [89]. In the latter study, the probability of a wild female mating with a wild male was 22-fold more likely than mating with a standard OK SIR male (250 Gy) even when released within an apple tree.

An individual-based model was developed to study the effects of individual behaviours on the mating rate between wild females and sterile males, and on sterile–wild trap catch ratio [116]. The detailed model considered effects of population aggregation, of female selectivity, of female movement between mating calls, and of sterile mating asynchrony. The results suggested that sterile males may not be effective at mating with wild moths during springtime releases. This results from a combination of behaviours such as asynchrony in timing of mating between sterile and wild moths, which increases the percentage of wild females fertilized. Also, the modelling confirmed that trap catch ratios of sterile and wild moths may give little to no indication of changes in mating rates, in part because of errors arising from the small proportion of the population captured in pheromone traps [116].

Further errors may arise in estimating a ratio of sterile–wild moths at a site, depending upon the type of monitoring tool used (typically pheromone trap with 1 mg codlemone lure). When using wild females, a difference in results occurs. Such effects lead to overestimates of the local availability of sterile males and underestimates of the relative size of the wild population [91]. Efforts to improve assessment techniques for codling moth are discussed below (Section 3.8). 

### 3.4. Spatial Relationships and Adult Flight

The spatial relationships of codling moth populations are important for its management at three levels [117,118]: the landscape level, the orchard or garden, and within trees in immediate proximity to the mating site. At the landscape level, the service area of the B.C. programme was deemed to be isolated enough to prevent immigration of inseminated females, because it lay within a group of interior valleys within a major mountain range. By 1992, short- and long-term dispersal was already well described for adult *C. pomonella*, at up to 11 km [119], and the major reinfestation problem was identified as the uninterrupted orchard landscape at the Canada–US border at Osoyoos, and possibly some other points at the periphery, which were to be identified by intensive monitoring. At the orchard level, three other problems were foreseen: Transport of overwintered diapausing larvae on fruit bins; populations on noncommercial host trees in private gardens and very small orchards; and populations on abandoned pome fruit trees [9].

At the outset of moth release in 1994, it rapidly became clear that the varied terrain and land use in the B.C. interior, which is highly fractured into relatively small parcels, caused unanticipated problems. The original pilot region of some 500 ha of the Similkameen Valley was fairly contiguous, well mapped, and every pome fruit tree within it, whether commercial, private or abandoned, received appropriate treatment [34]. As one contrast, the program began without knowing the numbers of untreated host trees anywhere, which increased greatly over five years, contributing to abandonment of the eradication mandate [11]. Use of a geographic information system (GIS) was essential to the OK SIR program [9], but no geodatabase existed for the B.C. interior and any related technologies [120] were, in fact, abandoned by 1996. A major research, development, and implementation project was required to develop the first integrated GIS of any kind in the region [121]. From 2000 on, it enabled the site-specific deployment of resources such as sterile moths. The identification and treatment of non-orchard sites was reduced from everywhere to sites within a “buffer zone” of 200 m of orchards (twice the estimated maximal movement distance of wild mated females), so as to reduce immediate costs. The problems of long-term source populations beyond 200 m distances, and associated reinfestation, were thereafter ignored and their solutions were reduced to educational information and requests for voluntary tree removals, as discussed below.

The creation of a GIS also enabled analyses of structural features of the landscape and of key orchard attributes related to efficacy of the OK SIR program, which confirmed empirical experience. For example, increased numbers of wild moths were associated with tree height, basal diameter, sites with high slope values, especially if south-facing, but with lower numbers in high-density orchards [122]. More complex studies [123] revealed similarly that tree basal diameter (tree age and potential suitability as moth habitat), as well as within-row tree spacing, and elevation above lake level, were consistently related to wild moth count and to proportions of wild and sterile moths captured (overflooding ratio). Wild moth counts were highest: (1) In traditional orchards with large trees; (2) in low elevation sites; (3) in orchards facing north; and (4) were consistently higher in different regions of the study than in others [124]. Some findings are explained by the greatly reduced numbers of codling moth larvae overwintering in modern high-density plantings in comparison with large, old trees [71,125,126], and by the critical importance of such habitat to the persistence of moth populations [127,128]. The OK SIR program has long recognised that large, older, trees, particularly with highly fissured bark such as pear, are associated with high-risk sites, having very persistent wild moth populations [2]. A different comparison of the presence of *C. pomonella* across different geographic areas during four years, 2010–2013, revealed that both moth presence in traps and fruit damage decreased significantly as orchard size increased [129].

These results of GIS analyses concur with landscape studies, discussed below, which indicate that codling moth management should be active in areas larger than single small orchards, suggested as >16 ha and above in size [117]. The latter effect appears to be related to the movement of codling moths across the landscape and within orchards, as determined in field studies in a mixed landscape of 895 ha [48,130]. Mark–recapture experiments with externally marked sterile moths showed the moths to be highly dispersive, with movements of at least one kilometer across the study area, trace movements of up to 3 km, effects of open versus closed canopies, of mild slopes, arising from proximity to small woodlots, and a limited ability of the moths to cross open terrain [48]. Prior workers had also regularly trapped insects carried by wind from neighbouring orchards [34] or from wild trees up to a 1.5 km distance [30,109]. In B.C., the movement of sterile marked *C. pomonella* was interrupted by many features, but linked in some cases by tree lines [130]. Our studies, as cited or unpublished [71], have convinced us that the sterile moths can be highly dispersive in comparison with wild moths, and may even respond differently to borders and edges. Hedgerows and windbreaks were found to have a significant effect on intra-orchard distribution of larvae arising from wild codling moths in France [117,131]. After modelling the B.C. data, recommendations were made for the most effective deployment of sterile moths on the downwind edge of orchards, and it was suggested that the edge trees act as barriers to flight over the orchard boundaries [132]. The model suggested that dispersal distances in landscapes with scattered host trees are likely higher than within apple orchards and probably dependent on the flight behaviour of males and females and wind conditions [132]. Studies in Chile similarly found marked differences in movement distances and rates between the within-orchard and extra-orchard populations, the latter being much greater [133].

Many of the results of movement and modelling studies have agreed with prior analyses of dispersal studies using mark–release–recapture, suggestive of a pest with a median dispersal distance <500 m. In laboratory studies, wild populations are composed mainly of individuals with a flight capacity of <100 m, but also having a critical proportion capable of flight distances of several kilometres [79,134,135,136]. Similar laboratory assessments have shown that flights of at least 7–10 km are more common in mass-reared program moths than they are in wild moths [93]. The modelling predictions [132] were also validated in field studies of codling moth larval density using molecular or immunological techniques in France [131,137] and Chile [133]. For example, females mainly clustered their eggs on contiguous trees along orchard borders [137]. One complication for comparison of studies is the increased fragmentation of the B.C. landscape, caused by apple growers reducing orchards and replanting with grape and sweet cherry, as well as reduced orchard size owing to changes in land use and residential development. This has resulted in a very different spatial environment than in the pilot studies of SIT by Proverbs and American counterparts in the 1970s, or than in less heterogeneous regions with large orchards, as occur in some fruit production areas [35]. In summary, the inherent capacity to fly and disperse would not appear to hinder the capacity of sterile moths to interact with wild populations. The uniform mode of moth release by ATV that is used presently may be unnecessary for success, as shown in pilot studies using single point releases [17,48,107].

### 3.5. Reinfestation and Ecological Factors Acting at Low Population Levels

Reinfestation of a site can occur via short-distance dispersal from neighbouring sources of untreated codling moth, or via long-distance dispersal from small numbers of wild moths in the airstream or carried on containers. Given the rates of increase observed in local orchards of 60–100-fold per year, control of immigration is critical [30,34]. The most successful strategy employed at the orchard-urban interface with private lands was host tree removal [11]. Incentives were given to remove and replant with nonhost plants, particularly in “buffer zone” sites within 200 m of commercial orchards (Section 3.4). The movement of diapausing *C. pomonella* on wooden containers or ladders was a known threat to any SIT programme [138], and Higbee et al. [139] showed that significant populations of codling moth entered bins during the time that they were in the orchard before harvest, such that deployment for only six weeks, in orchards with levels of moth infestation common in B.C., resulted in >3 to >12 larvae per bin. At the time, the apple industry shuffled annually some 500,000 wooden fruit bins throughout the service area [2]. After 2001, short-lived efforts were made by some packing houses to restrict bins moving from “dirty” to “clean” parts of the service area, or to delay their deployment until after the spring generation of codling moth had flown. Despite this awareness, a study in 895 ha of the Similkameen Valley, 2001–2003, concluded that the major source of reinfestation during the period was most likely from the movement of diapausing larvae associated with wooden bins [107]. We recently observed a similar pattern of reinfestation and spread of codling moth [56,71] following the importation of wooden bins into the same Valley from outside the service area in 2016 [71].

The control of diapausing codling moth in wooden bins was attempted using entomopathogenic nematodes [140] or with glacial acetic acid [141], both of which are compatible with the certified organic practices employed by many B.C. growers. The former method was successful at removing 80% of larvae in pilot trials, but very labour-intensive. Similarly, the use of glacial acetic acid was deemed to be neither practical nor economical for wooden bins. Neither practice was implemented. Recommendations were made since 2000 for an industry-wide transition to plastic apple bins which harbour far fewer, if any, overwintering larvae, and are phytosanitary on other grounds [2]. Economic and other constraints have limited their adoption to relatively low levels.

Once extremely low levels of codling moth were reached in the Similkameen Valley and elsewhere (e.g., [107]), an assessment and understanding of the population dynamics at the levels of individual sites and trees became very important (Section 3.8). For within-site habitats, including single trees or small groups, a statistical analysis of trap catches [123] suggested that local infestations were a more likely cause of wild moth persistence at a trap or site rather than by immigration or importation on fruit bins, ladders, tree props, etc. Such evidence of small local infestations was found within the pilot project of 2001–2003, when using twice weekly monitoring and sterile release in response to wild moth presence. The SIT was successful when monitoring twice weekly, treating only the immediate area around a trap (deployed at 2.5 per ha) after capture of one or more wild moths, and when ensuring a 40:1 ratio of sterile-wild moths in the trap(s) immediately afterwards [107].

The behaviour and ecology of overwintered diapausing larvae and of young adults on trees was also long known to be critical at low population densities [127,128]. Their occurrence and characteristics were studied in nearby Washington State [126], and in France [131,137]. The aggregation pheromone produced by cocoon-spinning larvae was found to attract or arrest other codling moth larvae, and was identified [142,143]. It was shown to enhance captures in the field [144], and attracted the prepupal parasitoid *Mastrus ridens* (formerly *ridibundis*) Gravenhorst (Hymenoptera: Ichneumonidae) [143]. A theoretical underpinning of the evolution of such behaviours linked them to ecological parameters [145]. The evidence is in agreement that male and female moths emerging from protected aggregations can mate more or less immediately, thus the chance of a sterile male successfully finding and mating is greatly decreased, and fruit damage can continue in localized “hot spots”. When a population is at a very low level, a population can be maintained by very few fertile moths and monitoring must be intense [109]. The release of sterile males must not only be synchronised spatially to such hot spots, but also temporally, as considered next.

### 3.6. Diapause and Climatic Effects on Competitiveness and Mating of Sterile Males

The main fruit-growing region of B.C. comprises the lower elevations of a group of inland valleys and plains in the rain shadow of coastal mountains. In common with many regions of pome-fruit production, it has hot dry summer and cold winter climates. A key problem in B.C. has always been the inactivity of sterile moths in spring. Poor early season moth performance occurred during some of the pilot research, and was predicted [2,10] to become worse as the OK SIR program began to deploy sterile moths in the northern part of the service area, which is cooler and wetter. Research into factors responsible for the pattern of sterile moth activity [115] has suggested that it may be due to four factors: (1) Greater mortality of sterile moths in spring owing to insecticide use or cooler temperatures, or that sterile moths may be (2) less responsive to pheromone traps in spring than summer, or (3) less dispersive and active under cool spring temperatures than they are in summer, or (4) trap saturation in summer may be misleading of reduced activity. It seems likely that most of the seasonal variation can be explained by a response to temperature during cool spring weather [69,115], and that such inactivity may be related to laboratory rearing conditions [115]. Radiation-induced impairment of the olfactory system was ruled out as a factor [82]. As a result, much attention was placed on changes in rearing and temperature acclimation (Section 3.1).

Whatever the cause, the spring (overwintered) generation of codling moth was never temporally available to the SIT, as shown by the analyses of ratios achieved at every trap, cited earlier (Section 3.3) [48,115]. Furthermore, a proportion of the larvae arising from spring generation moths are known to diapause immediately [132]. The proportion of the population entering diapause is triggered by temperatures and day length, usually varying between 10%–50%, and the diapause state can persist for up to two years [146]. In southern France, 6% of the codling moth larvae collected in spring went into diapause and emerged the following year rather than giving rise to a second adult generation [137]. Unfortunately, earlier researchers and program planners seem not to have considered the diapause condition or escape of parts of the population from SIT in summer as problems. The proportion of spring generation larvae entering diapause directly was assessed without comment (e.g., 10%–15% of the population) [17,30]. Despite the cold and wet conditions experienced in spring, the period of emergence of spring generation adults was regarded by Proverbs [30] as the most auspicious time for SIT, because of the unfavourable climate for moth reproduction and winds limiting dispersal; a virtually monogamous female moth mating less often than in the second (summer) generation; and a smaller population than in the summer generation, owing to larval mortality in winter. The reasons why Proverbs or others did not report problems with sterile moth activity during the spring generation, other than in periods of inclement weather, are unclear. Perhaps, it is due to some differences in mass rearing or colony health between then and now. Given the genetic component in univoltine diapause reported by Swiss workers, we hypothesise that some selection may occur under the SIT to worsen the problem in B.C., favouring survival of larvae that enter diapause immediately [48,71]. Regardless of cause, over time it became clear that the spring generation was more persistent than the summer generation under the SIT [11,48] and other techniques were required for success.

### 3.7. Area-Wide Management Techniques Employed in B.C.

A basic tenet of the SIT is that insect populations must be reduced to a low level before its use [66]. The OK SIR program relies upon the increased use of insecticides, and alternatives for certified organic farmers, to reduce moth populations in orchards when above certain thresholds [56,57]. The eradication concept targeted pre-release harvest damage levels of 0.1%–0.3%, and suggested that such population densities should be eradicated by SIT within three years [9]. In fact, in the pilot projects upon which the program was modelled [34], Proverbs emphasised that pre-release damage levels should be 0.05% or less at harvest. Later research concurred with the lower value as more likely for success [52]. For a number of reasons, even the 0.1%–0.3% strategy proved impossible to achieve across the service area [2]. Continued use of insecticides, or alternatives, was encouraged and extended via incentives to fruit-growers. Concurrently, from 2000–2014, the OK SIR program participated in large-scale tests of various mixes of SIT, and of alternative technologies, so as to reduce its reliance upon pesticides, to improve SIT, and to shorten time-lines, [2,3,47,50]. Elements, which became critical to its success, were added from local research in other integrated pest management (IPM) programmes.

The first area-wide technique employed was host plant removal, with teams of staff identifying and removing roadside or abandoned trees, and the creation of an urban team working with private citizens and public agencies. Legislation was added in many jurisdictions to prevent the use of host trees in residential plans, golf courses, etc., and some cities agreed to remove them from public spaces. Indeed, by 2000 the OK SIR program boasted of the removal of >200,000 abandoned and noncommercial host trees from its service area [2]. However, progress was slow; in 2010, 16 years after start-up, >33% of 35,000 host trees within 32,000 noncommercial sites in the service area were still active sites owing to the presence of *C. pomonella* [1]. Nevertheless, after visiting B.C., Kovaleski et al. [147] successfully employed host tree removal as the primary tactic for eradication of *C. pomonella* in Brazil and showed its value in reducing codling moth populations at a landscape level over time [62]. Host tree removal was also deemed to be very important in a codling moth area-wide management programme in the U.S.A. [113] and was highly recommended for such use in Chile [133].

A very different factor changed the urban and rural landscape after 1990, by its removal of large, old, trees. Government action to restore economic viability of the tree fruit industry led to subsidies to remove older pome fruit orchards with large trees, replacing them with small trees in high-density plantings, a programme which continues today. By 2003, some 2033 ha of apples were replanted, just when the area of apple and pome fruit was reducing from 7331 ha to 3395 ha [71], and has continued since then at some 100 ha per year (B. Jorde, B.C. Fruit Growers Association, pers. comm.). These area reductions were achieved in large part by removal of uneconomic older orchards and replanting with modern (smooth bark) high-density orchards as a requirement of funding. Both changes must have led to very significant reductions in overwintering habitat for larvae, and in moth survivorship [127,128], coincidental with program operations.

The development of pheromone-based mating disruption [35,148] and its integration with a number of approaches for population monitoring and reduction [52,125], notably cardboard tree banding, were very successful alternative technologies. Research showed that catches of wild moths and overwintering larvae declined faster in orchards receiving SIT with supplemental pheromone and banding than in insecticide-supplemented orchards [52]. The program used wax-treated cardboard banding extensively in 1999 for assessment of overwintered larvae across the service area and then adopted it for widespread use for population reduction in urban sites and limited use in problem orchards.

By the time it was proven that sterile–wild moth ratios were inadequate in springtime, it was apparent that trying to encourage continued heavy use of insecticides both defeated the purpose of the programme, and was not well accepted by growers [2]. A compromise of a single application of an insecticide in spring, in combination with a modified pheromone disruption treatment (1/2 recommended rate or 500 dispensers per ha), was adopted generally by the program in 2000–2004. During the 2000–2014 period, the dispensers for pheromone-based mating disruption (Isomate-C, Isomate C+, Isomate-CM LR, Shin-Etsu Chemical Co. Ltd., Tokyo, Japan) and associated traps (discussed below) were provided to all growers in various regions of the service area at no cost, or subsidised by various means. In some years the dispensers were also deployed in trees by the OK SIR program, as was cardboard tree banding on a smaller scale. Sales of mating disruption dispensers began with their use by organic growers in 1996–1999 (266–588 packages of 400 dispensers per year). This supplemental tactic was adopted massively by the OK SIR program in 2000–2004 (2436–3850 packages per year). It was again deployed in northern regions and problem sites where the use of sterile moths was withdrawn completely in 2012–2014, and subsequently, at commercial application rates [2,71,149]. After extensive field testing in orchards, Judd and Gardiner [52] went further and recommended a pheromone + tree banding programme applied in spring and release of sterile moths in summer only, as a successful and cost-effective programme, especially for certified organic sites. For some sites, the use of an organically certified broad-spectrum horticultural spray oil, acting as an ovicide, is also recommended to assist the poor early season performance of sterile males.

To assess program performance, an analysis of government and industry sales data, area-adjusted for changes in the service area, was conducted for 1991–2005 [149,150]. It showed that use of organothiophosphates, the insecticide group used for *C. pomonella*, was circa 0.95 kg per ha from 1991–1993, increasing to 1.3 kg per ha in 1995, decreasing again to 0.85 kg per ha by 1997 and 1998, before reducing to 0.45–0.55 kg per ha in 2000 to 2005. These four periods coincided with program initiation prior to 1993, insecticide “sanitation” 1994–1996, industry exhaustion in 1997–1998 after 3–5 years of intensive insecticide use, decreasing to the lowest use of insecticides in 2002 owing to substitution of mating disruption products in the prior three years. Interestingly, a similar analysis was conducted at the time in neighbouring Washington State, USA, which reported the same pattern of reductions in insecticide use as a result of adoption of mating disruption and of changes in the insecticides being applied [71] (Jay Brunner, Washington State University, personal comm.).

The impact of biological control agents on codling moth is often not recognised in commercial production areas. Proverbs [17] observed that >40% of overwintering larvae were commonly destroyed by *Ascogaster quadridentata* Wesmael (Hymenoptera: Braconidae) in insecticide-free orchards, and noted its absence from treated sites. From the beginning, the integration of biological controls within a codling moth SIT programme was considered. In particular, the possible use by parasitoids, *Trichogramma platneri* Nagarkatti (Hymenoptera: Trichogrammatidae), of the viable and nonviable eggs deposited by sterile codling moth females [151,152,153]. Field releases in apple orchards produced a significant decrease in codling moth damage [154] and related work has shown that the combined use of egg parasitoids and SIT is a valuable approach (reviewed in Cossentine and Vincent [155]). Laboratory studies in Argentina were similarly promising with other egg parasitoids of *Trichogramma* spp. [156]. Other studies have examined the potential for increased use of parasitoids through understanding of the kairomones used by codling moth larvae [142,144], in the host-finding of codling moth eggs by the parasitoid *A. quadridentata* [157], and of the sex pheromone of the latter [158].

The continued integration of novel methods for management of *C. pomonella* was tested at different times, notably the use of a pheromone-based attract-and-kill strategy [71,159] {Judd, 1994–2019 #11589}, which was not implemented. Similarly, combinations of tactics with SIT were tested successfully in large pilot trials (e.g., [107]) over the years, but were not usually implemented further [2], as discussed below.

### 3.8. Assessment of Insect Populations and Damage, Program Results, and Response Time

Both Knipling [66] and Proverbs [15] identified the assessment of the natural population density and its rate of increase to be critical factors for the SIT to work, particularly at low levels of abundance. However, the law of diminishing returns applies to detection costs in large projects, which increase as populations diminish [160]. Proverbs relied upon two methods: Within-orchard counting of proportion of fruits, including windfalls, injured by or containing larvae after each generation, at a very high sampling intensity, and the weekly pheromone trapping of marked sterile and unmarked wild moths. To these, the use of cardboard tree bands as a measure of larval density on trees [125] has been added. The latter can be more sensitive for finding populations of *C. pomonella* than damage surveys, and is less demanding in timing than other methods [52].

Harvest assessments have been a mainstay of the program since its inception, and in pilot studies apples from every bin, or fruit from every second tree, were examined so as to detect local infestations (e.g., [113]). For an operational SIT programme in fruit orchards, such an effort is required annually for all of the sites. It is extremely difficult to schedule the optimal time for different cultivars, and often simultaneously in many orchards across the service area. Instead, a statistical model was created from the results of program-wide trap captures, and of damage from *C. pomonella*, over two years. It was analysed using an empirical Bayesian model and a batch sequential sampling scheme. A method was then developed to optimize the time spent on harvest inspections by focusing on sites of greatest infestation potential according to damage in the prior year and wild moth counts in the current season [161]. A simplified technique was delivered for operational use, using stratified sampling with priority given to wild moth counts in the current season [162]. However, simulation studies showed that the number of orchard samples could only be reduced at best to 72% of sites, owing to the variability observed in the results [162].

The development of traps for monitoring populations of codling moth, and their relationship with fruit damage, has been important in entomological research in B.C. for >50 years. At first, by Harold F. Madsen and collaborators in some ten articles from several countries (citations in [59,163,164]), and continuing today (present article; [71]). Consequently, sex pheromone traps (1 mg lure of codlemone, or 10 mg lure under mating disruption) have been the method most often employed by the OK SIR program for assessment. They are effective in monitoring the general population levels, and for indicating codling moth reinfestation or build-up at very low population levels. At the time, Proverbs did not deem them to be satisfactory for establishing exact sites of infestation within an orchard, or of population density at very low levels, probably because of the aggregated nature of wild codling moth populations (e.g., [34]). More recently, when populations were very low (fewer than three moth captures per trap week) and at deployment rates of 2.5 per ha, they were used successfully for targeting release of sterile males within 0.5 ha around any positive trap (e.g., [11,48,71,107]). In addition, a treatment threshold of trap captures of two wild males for two consecutive weeks [59], has been consistently employed within individual orchards and the OK SIR program [57] for almost 50 years and has a proven relationship to a damaging population level in many regions of North America.

A series of incremental improvements in lures have been made, following the discovery of a host kairomone derived from pear (pear ester, ethyl-(E,Z)-2,4-decadienoate). In combination with either acetic acid or sex pheromone [165], it increases the sensitivity and attractiveness of lures to both sexes, but particularly females, perhaps by eliciting food-finding behaviour [165,166,167]. Theoretically, estimates of female density and of mating status should provide a better measure than male trap counts of any risk of fruit damage and of the effects of the SIT. The promising use of kairomones and other female attractants [168] for this purpose needs to be explored in more detail. In all likelihood, accurate codling moth monitoring based on female lures will require higher trap densities because the active space of these semiochemicals is often shorter than are pheromone signals [71]. Their use may require increased effort and costs but, in reality, increased trap densities and fine-grained monitoring are needed to adequately address the sampling inefficiencies that one trap per ha provides when populations are highly aggregated.

The use of pheromone traps alone should not be relied upon [34,38,116], and on occasion it is necessary to verify trap counts using methods more appropriate for the assessment of absolute population densities (e.g., fruit inspection or cardboard bands). When employing sterile moth release, two particular problems with traps have been identified in our cited and uncited [71] work. The first is trap saturation, which arises when sterile–wild ratios are high, creating difficulty in identifying one or a few wild moths in a clogged trap. As an example, in 2008 we monitored 263 properties in the Similkameen Valley and only 18 were found to have any wild moths seasonally. This occurred using high rates of sterile moth release by the OK SIR program, and at a trapping intensity of one per ha. In spring of the following year, sterile releases were discontinued so as to treat only where necessary, trapping intensity was increased to 2.5 per ha, and wild moths were caught in 53 properties [71]. The latter overwintered moths resulted from populations in the same sites in the year before, and mere changes in trapping and an absence of saturation provided an almost three-fold increase in detection. A second trapping problem is due to the dispersive and local nature of movement of sterile or wild moths, respectively, which provides an imbalance in true sterile–wild ratios in traps at different locations, depending in part upon topography, edges, or barriers such as open spaces. The propensity for flight, and highly dispersive nature, of mass-reared codling moths means that they likely encounter synthetic pheromone plumes more frequently than the relatively sedentary and aggregated wild moths. This likely occurs regardless of the length of pheromone reach of any synthetic lure used [169]. The resulting problem of inflated overflooding ratios, relative to true mating ratios, was discussed in Section 3.3.

All large SIT programmes, including the OK SIR program, have a related challenge of rapidly interpreting and responding to trap counts or other signs of infestation in hot spots. Large SIT projects often find it easier to have routine schedules of monitoring, and of release of large numbers of sterile insects, using a service model akin to that used by other public utilities (discussed in Section 4). This type of service model has been regularly associated with trap saturation and with poor detection in B.C. [2,48,71]. It was exacerbated in years when traps were placed in only a fraction of orchards [2,53]. In practice, strategies of high monitoring and of low release to sites, as and when required, have proven to be the most effective and economical in regions with low moth populations such as the Similkameen Valley [71,107]. The latter strategy provided a net reduction of sterile moths in low-risk sites, permitting releases in higher numbers in problem sites, and was determined to be the most sustainable in the long-term [2,47,48,49,63,107].

One early study [53] revealed that the practice of averaging measurements at a program level was responsible for very serious errors of population interpretation and of eradication status. At the time, combined averages from all sites for wild moth catches, of overflooding ratios, and of percentage of orchards with no damage at harvest, were calculated rather than the results being examined at the site-specific level. The use of more than one assessment method has also been required at times, as when 91% of orchards showed no damage in 1997 and the eradication of codling moth from a third of the service area was optimistically predicted by the end of 1999 [45] but did not occur. Subsequent analysis at the orchard level revealed that 70% of sites were trapping wild moths in 1998 and cardboard tree banding detected overwintered larvae at harvest in 1999 in >15% of orchards sampled [2,3,71].

Knipling [66] stressed the need for accurate information on crop losses and on costs of alternative control measures in comparison with rearing and release for SIT. Numerous consultants have prepared assessments of the economics of the program, or business case studies (e.g., [16,65]), since before its inception. Such business reports, as well as a study of pesticide use and grower attitudes [150], are mostly beyond the scope of the present review. However, two major studies were commissioned to examine the sustainability of the program post-2005, by which date a steady state of low-cost maintenance was required to exist across the entire service area. It was defined as equivalent to the very low population of wild moths found in the Similkameen Valley, circa 2000 (see [1,107]). The first made a comprehensive examination of the economics of pheromone-based mating disruption and of SIT, or combinations thereof, and the results were used as the basis for planning for many years [63,64]. The second examined the key technical questions to resolve for a least cost-effective programme using SIT [48]. Ten recommendations were made with respect to research needs and to resolve key operational questions; those still relevant are given below (Section 3.10 and Section 4).

### 3.9. Other Pests

Secondary pests, those arising from the adoption of area-wide techniques and reduction of broad-spectrum insecticides, have long been identified as a concern resulting from any use of the SIT for codling moth [17,110]. Early financial studies estimated that the addition of one additional chemical spray on half of the orchards would remove any overall financial benefits of the program [2,41]. It was also foreseen from early SIT pilot studies that, in the absence of insecticides for control of *C. pomonella*, leafrollers (Lepidoptera: Tortricidae) can build up to damaging numbers [34,38]. The eradication plans [9,54] suggested the development of supplementary IPM programmes for them, as and when they occurred. Actual field experience since 1994 has shown a rise in the importance of leafroller moths [2] and a now serious concern with eyespotted budmoth, *Spilonota ocellana* (Denis and Schiffermüller) (Lepidoptera: Tortricidae). Research on this group is continuing and has elucidated critical features of the biology of the latter [170,171], as well as the development of kairomone-based lures and traps [172,173] and of possible pheromonal methods of control [174].

The complexity of tree fruit production systems means that an insecticide applied for one insect may control another. In B.C., the requirement for any insecticide use having complementary activity on codling moth, such as for a new invasive insect, would reduce the rationale for, and economic viability of, the program. One problem was foreseen, i.e., the anticipated invasion of the apple maggot, *Rhagoletis pomonella* (Walsh) (Diptera: Tephritidae), which slowly crossed the neighbouring Washington State, USA, from 1980 onwards. Despite vigorous attempts to slow its spread [175,176], adults were detected near the centre of the program service area in 2015 and 2016. In many parts of Canada, apple maggot and codling moth have been comanaged because their populations can often be controlled economically using a single insecticide application [177]. In some cases, both can be reduced to undetectable levels and require only border treatments of orchards [178,179]. Another invasive insect pest of pome fruits, the brown marmorated stink bug, *Halyomorpha halys* Stål (Hemiptera: Pentatomidae) was recently detected throughout the program area [180]. It may also require insecticidal control for a period, which would render the release of sterile moths uneconomic or unnecessary.

Finally, it can be argued that the significant reduction of broad-spectrum insecticide use in orchards, which occurred until at least 2005 [149] and in northern regions until at least 2014, may have enabled the rapid spread of an invasive, trunk-boring, apple clearwing moth, *Synanthedon myopaeformis* (Borkhausen) (Lepidoptera: Sesiidae). It was found for the first time in apple orchards in the southern region of the service area in 2006 [181] and now occurs throughout the program area [71]. It has been the subject of research into its biology [71], of control by entomopathogenic fungi [182], and its major sex pheromone component has been identified [183]. The synthetic pheromone was sufficiently attractive for use in detection surveys and possible development of pheromone-based controls. One study examined the costs and benefits of possible mass trapping as a control method [184]. It concluded that there was a significant by-catch of nontarget beneficial insects and a concern with the associated loss of ecosystem services.

### 3.10. Future Research

Some 15 years ago, we suggested necessary research areas when using SIT and supplementary technologies for codling moth management [11,48,50], and these have changed little since then:

- Development of assays for measurement of critical attributes of field performance of mass-reared sterile moths and related assays for quality control in the rearing facility.
- Improved understanding of the effect of releasing sterile females together with males and the positive or negative effect of females in disrupting wild or sterile male orientation, or in acting as sperm sinks for wild or sterile males, particularly if future genetic methods permit single-sex releases.
- Knowledge of the ecology of wild moths at very low population levels or under an SIT programme, and of the cause of the local aggregations and persistent populations observed regularly in program operations [56].
- Knowledge of dispersal and movement of sterile moths in fragmented landscapes and how sterile and wild moths react to cues within their environment that lead to population aggregations. Sterile moths must find and act on aggregated populations of wild moths.
- When evaluating systems for inducing sterility, such as genetically modified insects, or novel moth delivery systems like drones, is there an increased interaction with wild moths or in mating success of sterile moths, relative to other systems? Especially at low population levels, when finding aggregations is critical?
- Population genetics and molecular tools suitable for the determination of origin of trapped moths (mass-reared or wild populations), any degree of population mixing, and identification of F1 moths originating from mass-reared colony crosses.
- Knowledge of the degree to which diapause and voltinism may affect the management of codling moth populations and be minimised by appropriate strategies of rearing or release. Can the SIT be altered to function in a cost-effective manner in regions with both cold and hot seasons, or is it best confined to warm climate regions, as are most of the SIT programs operating presently?
- Kairomone or low-dose pheromone lures and automated trapping systems for delineation of local infestations and improved measures of populations, and sex ratio, for sterile and wild moths [165,166].
- Introduction or augmentation of biological control agents, such as *Mastrus* spp. (Ichneumonidae), for population reduction in noncommercial hosts or in otherwise untreated sites [185,186].

## 4. Discussion and Recommendations

For the SIT of codling moth in B.C., a strategy of releasing mass-reared sterile moths has been found to work well under certain conditions of low population density, and using specific operating procedures. These must include the correct population monitoring, release frequency, radiation dose, handling, and the management of neighbouring untreated sites [71,107]. In the early 2000s, the incremental cost of employing SIT was estimated to be slightly more annually than was pheromone-based mating disruption. But only when each was applied together with supplementary technologies over a large area, and excluding all capital costs associated with the SIT (mass-rearing facility, Cobalt^60^ source, etc.) [63,64]. The strengths of SIT, for *C. pomonella*, are that the method is now relatively well-understood and works well as part of an overall, long-term program, but more slowly than expected. It is able to eliminate codling moth from a localised area, so removing a need for control in many places in most years, and low populations can be driven close to undetectable levels.

The weaknesses of SIT for codling moth in B.C. are that it requires very low populations for success, eradication of established populations has not proven possible, and timelines towards goals cannot be predicted reliably. A density-dependent “crash” of populations, predicted in some insects under SIT at low densities [66,187,188], has never occurred in codling moth populations in B.C. Proverbs remarked, and it remains unclear today, as to “whether the inability to suppress codling moth populations below a certain level by the SIT is due solely to immigration or to an increase of reproductive rate at low population densities” [38]. Mathematical modelling is often used to understand the potential effects of various SIT strategies [187,188], but is of no value at present for explaining codling moth SIT. Little attempt has been made to build upon 25 years of field experience. No model has yet incorporated the effects of missing major parts of the spring generation of wild moths due to weather or asynchrony in mating, or of the total population owing to diapause immediately after the spring generation, of very low levels of reinfestation, nor has aggregation been well handled in the models to date [187,189]. One recent model [190] used values for some biological parameters of at least fourfold those estimated in B.C. [91,116,132], with consequent effects upon the results.

Unfortunately, the economies of scale in rearing and sterilising moths are currently poor, so large projects are required [2,63,64]. In many pome fruit production regions, alternatives are available in the form of insecticides, or mating disruption, which are scalable without large capital costs and can be purchased as required. Currently, there are more alternatives for control of codling moth available to certified organic apple growers than for any other moth in horticulture. Mating disruption and some other alternatives may control two or more insects at once [191]. Both factors were reported as important in the demise of an SIT programme for codling moth that operated as a private business in South Africa, despite positive results [60]. For B.C., the expense of rearing requires an area-wide programme, foreign sales, and general taxation or other revenues or subsidies, to be cost-effective [2,4,54]. If considering international facility-sharing, then transportation costs, flight schedules, weather, and flight availability have been problems, and shipping and application costs are expensive on a small scale [61].

Space does not permit the discussion of the many less critical items for which the OK SIR program has requested research or advice. At this point, it is useful to note the exploration of methods to prolong life of the costly Cobalt^60^ source (Gammacell 220, Nordion, Canada) and its similarly costly disposal, periodically, as well as its replacement with a less expensive X-ray device [192]. In our opinion, the current method of sterilization of codling moths poses the least problem in use of the SIT. Consequently, the value of any novel method lies in the costs and benefits of the performance of sterile moths relative to the wild moths, particularly at low population levels, and in related ecological and behavioural processes [15]. The development of new genetic techniques for production of sterile moths, and potential for release of single sex rather than mixed-sex moths, are exciting for future use of the SIT and for lowering costs [188,193], with the above caveats. The authors were involved in extensive discussions (2003–2005) between the OK SIR program and a British company seeking to purchase the mass rearing facility and to use recombinant DNA techniques for rearing sterile male codling moths.

To be cost-effective, an SIT programme for codling moth requires an area-wide approach, a complex infrastructure, and the cooperation of communities and groups, which have all been at times difficult to sustain [2,3,5,7,35,48,51,53,63], as in other such projects [12,35,194]. Entomologists with experience of large area-wide and SIT programmes have identified the key factors associated with project success [15,38,66,100,194,195,196]. Two of these are organizational and were not yet discussed: An organisation capable of effecting rapid changes in all phases of a large scale operation; and systems for monitoring programme effectiveness and making rapid changes in tactics. The OK SIR program has struggled with both types of issues, perhaps because the attitude adopted from the outset in 1992 was that all necessary research on codling moth SIT was complete. The governing board did not require a research plan and has rarely included research or methods development within the budget. The annual provision of detailed results, and any technical review, ceased in 2014. Consequently, most of the program functions and the core technologies employed in the SIT component have changed little in 25 years.

Several strategies of SIT in combination with different management tactics were tested in many “implementation trials” or pilot trials (e.g., [107]) over the years. Usually, these were not employed subsequently [2]. Primarily, because of challenges in replacing the current low-investment, local government public utility service model with a nimble field-responsive model. An effective service model should be capable of responding to infestation signals, such as trap captures, within a few days and altering release rates immediately in response. By contrast, from 2015 onwards, the program halved the frequency of moth delivery and reduced the release rate of moths, which together cut the number released per ha to one quarter of that used earlier [2,4]. In the same way, useful changes to the rearing process, or techniques of acclimation, were identified but not pursued, owing to limitations of funding or of a rearing facility built for one purpose [2]. One example was mass rearing using fluctuating temperatures, which proved impossible. Even if possible, one study found that gains in performance seen when using low temperature acclimated moths under cool conditions in the wild came at a cost to performance under warmer conditions [94], requiring further research.

More promisingly, the advent of hand-held computers, instant mapping of results by a GIS, and advances such as automated trapping technology and aerial release by drones may be able to provide the necessary speed and flexibility required, and rearing facilities elsewhere are being built with more flexible or multi-use designs.

In order to provide a sustainable and effective model of area-wide management of codling moth using SIT, we make the following recommendations:

- Replacement of a mass-rearing colony every two years, using a total colony replacement because of the likely selection against introduced genes if only augmenting wild moths to an existing colony. Development should be a continuous process acting in parallel with regular mass rearing so as to sustain service, because in our experience it can require two years to multiply to the necessary size for use.
- Development of assays for measurement of sterile moths by attributes that are critical for field performance and their linkage to assays that can be performed in a laboratory or rearing facility. Comparison of the quality of sterile colony moths against wild moths is critical in field tests of mark–recapture and mating, and is required when developing a new colony. Testing can more easily occur in a region with natural levels of wild moth populations than where an SIT programme is active.
- A recommended radiation dose for *C. pomonella* of 250 Gy. Use of reduced doses has not proven to improve activity and mating competitiveness of sterile moths during spring, critical to success against the overwintered generation of wild moths, but may cause problems of interpretation.
- A high intensity trap monitoring effort in spring, using at least 2.5 traps per ha, containing low load pheromone lures (10 to 100 micrograms), female kairomones, or related attractants [168] to identify local aggregations or “hot spots” [165], so as to deploy additional targeted or control efforts during the critical spring generation.
- The frequency of sterile moth release should be twice weekly, or more, per site. The most successful periods for the OK SIR program to date, 1995–2003 [2], and for pilot low-release projects [71,107], occurred when applying 4000 moths per ha (or less according to trap captures, infestation history, etc.), twice each week, for a total of 8000 moths per ha week. A high frequency is most useful in inclement weather, as in spring. Unstable weather, especially wind and rain, reduces the dispersal and flight of moths from the wet ground cover where they are deposited. If sterile moth supply is limited, our experience has shown that it is better to release twice weekly than once weekly. It is easier to ensure high sterile–wild ratios by targeting twice weekly [71,107], or daily [34], according to trap captures, than weekly.
- Use supplementary technologies where necessary to reduce wild moth populations in spring. Presently, the program requires supplementary use of insecticide or pheromone-based mating disruption products, and the use of multispecies products [197] is preferable. Use of supplements in spring, and limiting the release of sterile moths to the summer period only, is cost-effective [52,63].
- A strong and continued management effort is essential in non-commercial or urban sites to reduce populations on an area-wide basis, to a minimum of 200 m, preferably up to 1.5 km, from orchards.
- Replace annual harvest damage estimates by assessment of overwintering larval densities, which provides a direct measure of population density and of programme progress. Use a systematic program of cardboard banding applied to all orchards over a two or three year period; at minimum, banding should be in all problem orchards, each generation.
- At intervals, assessments of effectiveness must include pesticide use data for a representative sample of sites, in order to understand the control effort. Otherwise, it is impossible to assess the impact of any strategy on moth populations, nor to assess economic and environmental costs and benefits.
- Mathematical models should be designed with consideration to field experiences, including the avoidance of the effect of SIT where local aggregations of larvae result in immediate mating of wild moths; or on major parts of the spring generation of wild moths due to weather or asynchrony in mating; or on the summer generation owing to immediate diapause of a portion of the spring cohort; and with appropriate values for biological parameters.

## Supplementary Materials

The following are available online at https://www.mdpi.com/2075-4450/10/9/292/s1, Table S1: Locations of selected documents at internet sources on 15 August 2019.
