# Supplementary material for: Twenty-five Years of Research Experience with the Sterile Insect Technique and Area-Wide Management of Codling Moth, Cydia pomonella (L.), in Canada
Source: Insects. 2019 Sep 10;10(9):292. doi: 10.3390/insects10090292 (PMC6780149; doi:10.3390/insects10090292)
Supplement: Supplementary file 1 [file insects-10-00292-s001.pdf]

insects

MDPI

Supplementary Material

Twenty-five Years of Research Experience with the Sterile Insect Technique and Area-Wide Management of Codling Moth, *Cydia pomonella* (L.), in Canada

Howard M. A. Thistlewood [1,2,*] and Gary J. R. Judd [1]

[1] Summerland Research and Development Centre, Agriculture and Agri-Food Canada, Summerland, BC V0H 1Z0, Canada
[2] Department of Biology, University of British Columbia, 3333 University Way, Kelowna, BC V1V 1V7, Canada
* Correspondence: howard.thistlewood@Canada.ca; Tel.: +1-250-494-6419

Received: 12 July 2019; Accepted: 2 September 2019; Published: date

The Supplemental materials contain the locations of documents and reports which are also available directly from internet sources. Contact the authors for copies of other materials cited.

Table 1. Locations of selected documents at internet sources on 15 August 2019.

Reference	Location url
1.	http://www.Oksir.Org/wp-content/uploads/2016/07/sirguide042011final.Pdf
4.	http://www-naweb.iaea.org/nafa/ipc/Cara-Nelson.pdf
12.	http://www-naweb.iaea.org/nafa/ipc/public/ipc-sterile-insect-technique.html
35.	https://www.cabi.org/cabebooks/ebook/20083134928
45.	http://www-naweb.iaea.org/nafa/ipc/public/Area-Wide-Control-Insect-Pests-book.pdf
46.	http://www-naweb.iaea.org/nafa/ipc/public/Area-Wide-Control-Insect-Pests-book.pdf
47.	https://opdmc.org/wp-content/uploads/2019/01/2004OPDMC-Abstracts.pdf
55.	http://www.oksir.org/wp-content/uploads/2016/08/Strategic-Plan-12_June_2.pdf
56.	https://www.oksir.org/orchard-information/trap-viewer/
57.	https://www.oksir.org/orchard-information/codling-moth-maps/
58.	https://www.goodfruit.com/sterile-codling-moth-factory-a-funky-business/
62.	http://www-naweb.iaea.org/nafa/ipc/public/ipc-area-wide-control.html
65.	http://library-1.okanagan.bc.ca/vwebv/ocir/SIR_B_C_Analysis_Report.pdf
67.	http://www.fao.org/3/i1537e/i1537e00.htm
75.	http://www-naweb.iaea.org/nafa/ipc/public/ipc-codling-moth-sit.pdf
101.	http://www-naweb.iaea.org/nafa/ipc/public/EL_Lepidopterous_sterility_1971.pdf
106.	http://www-naweb.iaea.org/nafa/ipc/public/El_Computer_Models.pdf
120.	http://www-naweb.iaea.org/nafa/ipc/public/Sterile_Insect_Technique_book.pdf
129.	http://www.oksir.org/wp-content/uploads/2016/08/SIR_Report_PGill_June15_2014.pdf
187.	http://www-naweb.iaea.org/nafa/ipc/public/Sterile_Insect_Technique_book.pdf

© 2019 by the authors. Submitted for possible open access publication under the terms and conditions of the Creative Commons Attribution (CC BY) license (http://creativecommons.org/licenses/by/4.0/).
